# Challenges of pathogen inactivation in animal manure through anaerobic digestion: a short review

**DOI:** 10.1080/21655979.2021.2017717

**Published:** 2022-01-04

**Authors:** Min Lin, Aijie Wang, Lijuan Ren, Wei Qiao, Simon Mdondo Wandera, Renjie Dong

**Affiliations:** a College of Engineering, China Agricultural University, Beijing, China; b Key Laboratory of Environmental Biotechnology, Chinese Academy of Sciences, Beijing, China; c Department of Civil, Construction & Environmental Engineering, Jomo Kenyatta University of Agriculture & Technology, Nairobi, Kenya

**Keywords:** Animal manure, anaerobic digestion, pathogens, inactivation, influencing factors, bioenergy production

## Abstract

Animal manure is the main source of bioenergy production by anaerobic digestion (AD). However, the pathogenic bacteria in manure may pose a high risk to human health by contaminating the environment if not effectively inactivated during AD. Worldwide, more than 20,000 biogas plants are running for the treatment of animal manure. AD has been playing the important role in establishing a circular economy in the agricultural sector and may contribute to the United Nations sustainable development goal (UN SDG). Nevertheless, whether AD is a reliable approach for pathogens inactivation has been challenged. A comprehensive understanding of the coping mechanisms of pathogens with adverse conditions and the challenges of establishing the AD process to inactivate effectively pathogens are yet to be analyzed. In this review, the diversity and resistance of pathogens in animal manure are summarized. The efficiencies and the difficulties of their inactivations in AD are also analyzed. In particular, three forms of pathogens i.e. sporing-forming pathogens, viable but non-culturable (VBNC) pathogens, and persistent pathogens are discussed. The factors influencing the pathogens’ inactivation and AD efficiencies are analyzed. The trade-off between energy production and pathogens inactivation in an AD system was consequently pointed out. This review concluded that the development of anaerobic processes should meet the goals of high efficient bioenergy production and deep hygienization.

## 1. Introduction

The World Health Organization (WHO) ranked infectious diseases caused by pathogens including viruses, bacteria, fungi, and parasites as the second leading cause of death [1]. A major source of these pathogens is animal manure, which harbors pathogens, including disease-causing and non disease-causing pathogens. The use of animal manure as fertilizer to improve soil quality and fertility in agriculture has a long tradition. This causes the pathogen to spread from soil to water bodies, which becomes a potential source of environmental pollution and human infection [2]. It was reported by WHO that there were 600 million illnesses and 420,000 deaths caused by 31 foodborne pathogens globally in 2010 [3]. And during 2019, 25,866 cases of infection, 6164 hospitalizations, and 122 deaths were identified by the Foodborne Diseases Active Surveillance Network [4]. Considering safety and health, hygienization treatment for animal manure should be mandatory. Historically, this is an issue of widespread international concern. The control and destruction of pathogens can be achieved via biological, physical, and chemical methods or a combination of these. Composting and AD are the most common hygienization methods.

AD is a well-established technology for the treatment of various organic wastes and wastewater for bioenergy recovery, organic fertilizer production, and environmental protection. And it is in consistency with UN SDG No. 7 (Affordable and Clean Energy) which advocates for the reduction of waste through recycling and reuse [5]. As a part of bioengineering, AD contributes a bright future for the solution of the problems like resources, environment, and human health facing the world, and further provides sustainable agriculture with economic and social, and environmental benefits [6]. AD has been widely used in the treatment of animal manure. There are about 1347 million tonnes of animal manure produced annually throughout Europe and 1200 million tonnes of animal manure in the EU, and the theoretic biogas potential of manure was estimated at 26 and 23 billion m^3^ biomethane in Europe and the EU, respectively [7]. In the United States, there were more than 2100 biogas plants in 2017, of which 250 farm-based digestion plants used livestock manure [8]. In Europe, biogas production from agricultural waste, manure, and energy crops accounts for about 74% of the primary biogas energy output [9]. In China, there was 1.8 × 10^9^ t livestock manure in 2017, and the amount of livestock manure used for biogas production is 9.5 × 10^8^ t, and the biogas production potential is approximately 5.7 × 10^10^ m^3^ [10]. The substitution of biogas for fossil fuels is driving the sustainable development of many countries.

Meanwhile, the biosafety of the residues generated from the AD plants is the basis for the application of this technology. In some countries, the level of pathogens in anaerobic residues is regulated, and the application of AD effluent on land is permitted upon meeting the environmental quality requirements. It should be stressed that even the level of indicator pathogens is below the detection limit, this cannot be directly translated to the absence of potential pathogenic risk due to the occurrence of other pathogens [11]. It has been reported that most mesophilic biological treatment processes are not likely to reduce pathogen levels by 90%-99% [12]. What’s more, some pathogens can even multiply in the biogas plant environment [13]. Pathogens can be transported from manure-amended soils into water [14], and pathogen infections occurring by the fecal-oral route account for a large proportion in water environments. For a long time, the main focus has been on the efficiency of AD, but less on the controlling of pathogens. The inactivation of pathogens requires a high temperature and high volatile fatty acids (VFAs) environment, but the current anaerobic process is difficult to meet this high-stress environment in bioreactor due to the main goal of methane production in AD process. It is a challenge to achieve simultaneously the efficient methanogenesis and effective sterilization in AD.

Literature reviews about pathogens in animal manure tend to focus on the classification and description of pathogens, the methods involved in the control of pathogens, factors affecting pathogens deactivation in AD, and the mechanisms, kinetic models for pathogen inactivation, and so on. In this review, both disease-causing bacteria and indicative pathogens were discussed. The challenges of pathogen inactivation in animal manure through AD from tolerant pathogens and the limitation of the anaerobic process were discussed emphatically. By combining the inactivation efficiency of pathogens and the energy production of the AD process, this review aims to guide the practical design of AD systems.

## 2. Existence phenomenon of pathogens in animal manure

### 2.1. The diversity of pathogens in animal manure

The types of pathogens mainly include bacteria, viruses, parasites, and fungi. Pathogenic bacteria cause diseases by the production of toxins. *Bacillus, Clostridium, Escherichia, Staphylococcus*, and *Vibrio* can produce enterotoxins. However, some bacteria are nonpathogenic and therefore harmless. Bacterial pathogens from 31 genera in the virulence factor database (VFDB) (http://www.mgc.ac.cn/VFs/main.htm) are shown in Table 1 and the specific species that can cause diseases were summarized. This is helpful to strengthen the understanding of the pathogenicity of pathogens. And it will be more targeted to control pathogenic bacteria by distinguishing disease-causing bacteria. Viruses cause diseases by infecting cells and commandeering cell machinery to produce more viruses at a rapid rate. There are two main categories of parasites harmful to humans: pathogens that can cause parasitic diseases and vectors that can transmit diseases. Pathogenic fungi make people and other organisms sick even die.

**Table 1.** The main disease-causing bacteria

Bacteria (Genus)	Disease-causing bacteria (Species)
*Acinetobacter*	*A. baumannii, A. nosocomialis, A. pittii*
*Aeromonas*	*A. hydrophila, A. salmonicida, A. veronii, A. caviae*
*Anaplasma*	*A. centrale, A. marginale, A. phagocytophilum*
*Bacillus*	*B. anthracis, B. cereus*
*Bartonella*	*B. bacilliformis, B. quintana, B. henselae*
*Bordetella*	*B. pertussis, B. parapertussis, B. bronchiseptica*
*Brucella*	*B. melitensis, B. abortus, B. suis, B. canis.*
*Burkholderia*	*B. pseudomallei, B. mallei, B. cenocepacia, B cepacia*
*Campylobacter*	*C. jejuni*
*Chlamydia*	*C. trachomatis, C. pneumoniae*
*Clostridium*	*Cl. absonum, Cl. argentinense, Cl. barati, Cl. bifermentans, Cl. botulinum, Cl. butyricum, Cl. cadaveris, Cl. carnis, Cl. clostridioforme, Cl. chauvoei*, *Cl. difficile, Cl. fallax, Cl. glycolicum, Cl. haemolyticum, Cl. histolyticum*, *Cl. intestinalis, Cl. limosum, Cl. malenominatum, Cl. novyi, Cl. disporicum* *Cl. paraputrificum, Cl. perfringens, Cl. putrificum, Cl. septicum, Cl. sordellii, Cl. sporogenes, Cl. sunterminale, Cl. tertium, Cl. tetani*
*Corynebacterium*	*C. diphtheria, C. jeikeium*
*Coxiella*	*C. burnetii*
*Enterococcus*	*E. faecalis, E. faecium*
*Escherichia*	Enterotoxigenic *E. coli* (ETEC), Enteroinvasive *E. coli* (EIEC), Enteropathogenic *E. coli* (EPEC), Enterohemorrhagic *E. coli* (EHEC), Enteroaggregative *E. coli* (EAEC), Diffusely adherent *E. coli* (DAEC)
*Francisella*	*F. tularensis, F. novicida, F. philomiragia*
*Haemophilus*	*H. influenzae*
*Helicobacter*	*H. pylori*
*Klebsiella*	*K. pneumoniae*
*Legionella*	*L. longbeachae, L. pneumophila*
*Listeria*	*L. monocytogenes, L. ivanovii*
*Mycobacterium*	*M. tuberculosis, M. leprae, M. ulcerans*
*Neisseria*	*N. gonorrhoeae, N. meningitidis*
*Pseudomonas*	*P. aeruginosa*
*Rickettsia*	*R. rickettsii, R. conorii, R. prowazekii, R. typhi*
*Salmonella*	*S. typhimurium, S. typhi*
*Shigella*	*S. dysenteriae, S. flexneri, S. boydii, S. sonnei.*
*Staphylococcus*	*S. aureus, S. epidermidis*
*Streptococcus*	*S. pyogenes, S. agalactiae*
*Vibrio*	*V. cholerae, V. parahaemolyticus, V. vulnificus*
*Yersinia*	*Y. pestis, Y. pseudotuberculosis, Y. enterocolitica*

### 2.2 Resistance of pathogens in animal manure

**Figure 1.**
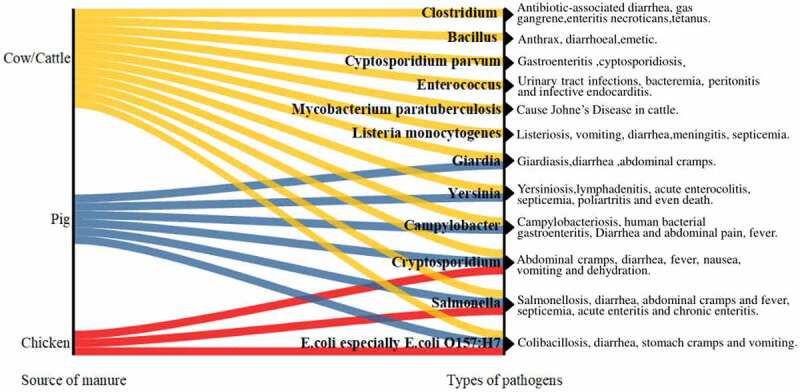
Examples of pathogenic microorganisms in different animal manures.

A large number of pathogenic bacteria exist in animal intestines, being able to adhere to the manure. Pathogenic bacteria species and contents in different kinds of animal wastes may be different. Take *Escherichia coli* (*E. coli)* as an example, in the cattle manure, chicken manure, and swine manure, their contents are 10^7^–10^8^ colony-forming unit (CFU)/g, 2.3 × 10^4^–2.3 × 10^7^ CFU/g, and 10^2^–10^6^ CFU/g, respectively [15–17]. Additional examples of pathogenic microorganisms likely to be found in manure are shown in Figure 1, and the likely diseases or symptoms are also presented. From Figure 1, there are more types of pathogens in cow manure, which may be related to the animal shape and intestinal characteristics. Among these pathogens, zoonotic pathogens should be paid more attention because zoonotic diseases can spread between animals and people and cause threats to human and animal health.

The physiological structures and characteristics of pathogens determine their resistance to adverse environmental conditions. Gram-negative bacteria are sensitive to high temperature and VFA levels and have low resistance against the stress environment due to their thin (single-layered) cell walls constituting only a lipid-rich outer membrane and a monolayer of peptidoglycan. Gram-positive bacteria have thick (multi-layered) cell walls constituting multi-layered peptidoglycan, which is the structure of the cell membrane and acts as a permeability barrier to prevent the toxic chemicals from penetrating the cell. In addition, spore-forming gram-positive bacteria has high heat resistance. In the normal environment, the spores can survive for many years [13]. Many protozoan parasites can also protect themselves from environmental stressors due to their ability to form cysts and oocysts [18]. Virus ribonucleic acid (RNA) and protein capsid are the main resistance mechanism.

Literature data about the efficiencies of AD process on pathogens inactivation in animal manure were summarized in Table 2. For *E. coli* and coliforms, some studies showed a good inactivation effect, and the content in the discharge was lower than the detection limit. However, some studies showed that pathogens as high as 10^2^–10^3^ most probable number (MPN)/g total solid (TS) or CFU/mL still exist in the discharge. For *Enterococcus* and *Clostridium perfringens*, which are difficult to kill, the log_10_ reduction is still low, mostly <2, even if they are in thermophilic condition.

**Table 2.** Literature data about the efficiency of AD process on pathogens inactivation in animal manure

Pathogen	Substrate	T (°C)	Operation mode	Initial count (CFU/g or mL)	Finial count (CFU/g or mL)	Log_10_ reduction	T_r_^a^/HRT (d)	Reference
*E. coli*	Dairy manure	25	Batch	6.5 × 10^7^	<10^2^	>6	60	[19]
*E. coli*	Dairy manure	35	Batch	3.6 × 10^5^	Below LOD^b^	3.6	62	[12]
*E. coli*	Cow manure	37	Batch	1.85 × 10^7^	<10^3^	4	41	[19]
*E. coli*	Cow manure	37	Batch	6 × 10^4^	Below LOD	4.94	6	[31]
*E. coli*	Chicken manure	37	Batch	2.3 × 10^7^	35	5.8	35	[20]
*E. coli*	Chicken manure	42	Batch	1.7 × 10^7^	27–34	5.7	9	[20]
*E. coli*	Dairy manure	52.5	Batch	2.5 × 10^7^	Below LOD	>7	3.5	[19]
*E. coli*	Chicken manure	55	Batch	10^6^	2–4	5.5	2 h	[16]
*E. coli*	Cow manure	55	Batch	6 × 10^4^	Below LOD	4.37	40 min	[31]
*E. coli*	Cow manure	70	Batch	6 × 10^4^	Below LOD	2.6	2 min	[31]
*E. coli*	Swine manure	24	Continuous	4.0 × 10^2^–5.8 × 10^5^		0.9–2.9	7,14	[17]
*E. coli*	Cow manure	33	Continuous	1 × 10^3^	3 × 10^2^	~0.5	55	[21]
*E. coli*	Cattle manure	45	Continuous		Below LOD	3	55	[21]
Coliforms	Swine manure	22	Batch	10^5^–10^6^	~10^3^	2–3	25	[22]
Coliforms	Cattle slurry	22	Batch	10^4^–10^5^	~10^2^	2–3	28	[22]
Coliforms	Swine manure	38	Batch	10^5^–10^6^	Below LOD	3–4	3	[22]
Coliforms	Cattle slurry	38	Batch	10^4^–10^5^	Below LOD	2–3	14	[22]
Coliforms	Swine manure	55	Batch	10^5^–10^6^	Below LOD	3–4	1	[22]
Coliforms	Cattle slurry	55	Batch	10^4^–10^5^	Below LOD	2–3	1	[22]
Coliforms	Cattle manure	33	Continuous	10^3^	10^2^	1	55	[21]
Coliforms	Cattle manure	45	Continuous	10^3^	10^2^	1	55	[21]
Total coliforms	Swine manure	24	Continuous	6.0 × 10^2^–1.5 × 10^6^		1.0–2.9	7,14	[17]
Fecal coliforms	Swine manure	24	Continuous	5.0 × 10^2^–1.6 × 10^6^		0.9–3	7,14	[17]
Fecal coliforms	Pig slurry/manure	39	Continuous	1.8 × 10^4^	2.0 × 10^3^	1.1–4.1	28	[33]
*Salmonella*	Swine manure	16	Batch	10^6^–10^7^	10–10^2^	5	60	[23]
*Salmonella*	Swine manure	22	Batch	10^6^–10^7^	10–10^2^	5	60	[23]
*Salmonella*	Dairy manure	35	Batch	7.4 × 10^3^	Below LOD	1.9	133	[24]
*Salmonella*	Swine manure	37	Batch	10^6^–10^7^	10–10^2^	5	3	[23]
*Salmonella*	Swine manure	24	Continuous	<10^2^-5.0 × 10^3^		0.9–1.4	7,14	[17]
*Enterococcus/Enterococci*	Cow manure	37	Batch	6.6 × 10^5^		3.13	15	[31]
*Enterococcus/Enterococci*	Cow manure	55	Batch	6.6 × 10^5^		1.7	2	[31]
*Enterococcus/Enterococci*	Cow manure	70	Batch	6.6 × 10^5^		1.77	1	[31]
*Enterococcus/Enterococci*	Pig manure	24	Continuous	1.8 × 10^4^–1.7 × 10^6^		0.6–1	7,14	[17]
*Enterococcus/Enterococci*	Cow manure	33	Continuous	10^6^–10^7^	10^4^	2–3	55	[21]
*Clostridium perfringens*	Cow manure	37	Batch	10^3^–10^4^		1.35	15	[31]
*Clostridium perfringens*	Cow manure	55	Batch	10^3^–10^4^		<1	2	[31]
*Clostridium perfringens*	Cow manure	70	Batch	10^3^–10^4^		<1	1	[31]
*Clostridium perfringens*	Swine manure	24	Continuous	<1 × 10^3^–3.7 × 10^6^		0–0.2	7,14	[17]
*Clostridium perfringens*	Pig slurry/manure	39	Continuous	5 × 10^4^	4.2 × 10^3^	1.08	28	[33]
*Clostridium perfringens*	Pig slurry	37–43	Continuous	8.9 × 10^4^	5 × 10^4^	0.25	40–56	[34]
*Clostridium perfringens*	Cattle manure	48	Continuous	1.9 × 10^5^	1 × 10^6^	−0.74	90	[34]

A, Tr, residence time of batch experiment; b, LOD, limit of detection.

## 3. Challenges of pathogens inactivation in the AD process

### 3.1. Selecting indicative pathogen

**Figure 2.**
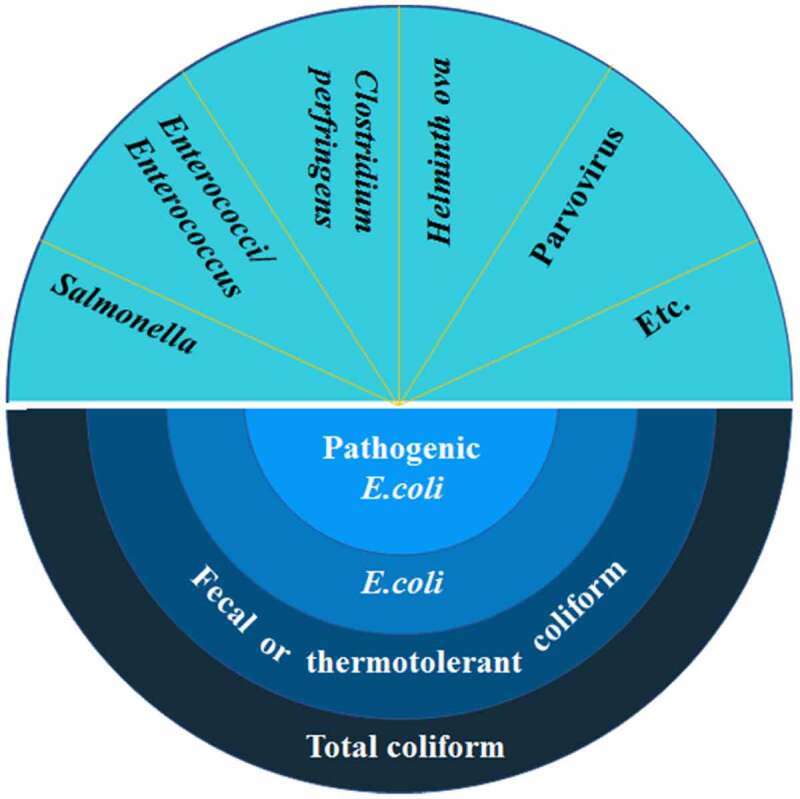
Common indicator bacteria.

Assessing the biosafety of anaerobic residues is essential for purpose of the application in the fields. However, there are many kinds of pathogens in anaerobic residues, and a number of pathogens are present in low concentrations. Detection of all of the pathogens may be challenging or time-consuming. Therefore, indicator bacteria are used to detect the possible presence of fecal pathogens and to indicate the effect of hygienic treatment of biowaste. The commonly used indicator bacteria are total coliforms, fecal coliforms, *E. coli, Enterococci, Salmonella*, and others (Figure 2). Due to their large numbers occurring naturally in human and animal intestinal tracts and the fact that they are easily detectable and countable, they are selected as pathogen indicators. It is important to note that indicator bacteria are not necessarily pathogenic bacteria. Indicator bacteria are the types of bacteria used to detect and estimate the level of fecal contamination of water. Their presence indicates contamination that could be pathogenic to humans thus is used to indicate the presence of a health risk.

Total coliforms, a group of aerobic or facultative anaerobic gram-negative budless bacillus, can ferment lactose at 37°C and produce acid and gas within 24 h. The group members include *Escherichia, Klebsiella, Enterobacter, Serratia, Citrobactera, Edwardsiella*. Fecal coliforms, also called thermotolerant coliforms, are a sub-group of total coliforms and distinguished from total coliform by the ability to ferment lactose at 44.5°C. *E. coli* and *Klebsiella* are the group members. *E. coli*, a sub-group of fecal coliforms, belongs to gram-negative bacteria. Some specific *E. coli* serotypes (i.e. O157:H7) are pathogenic to humans and animals and can cause severe diarrhea and septicemia. *Salmonella*, gram-negative, non-spore forming, aerobic, and facultative anaerobic organisms, belongs to the family of Enterobacteriaceae. *Enterococci* are suggested as the most suitable indicator bacteria to validate the hygienic treatment of biowaste in biogas plants. The genus *Enterococcus* is also called fecal streptococci. In addition, *Clostridium perfringens, Helminth ova*, and Parvovirus are also used to be indicator bacteria. But their standards are less common.

**Figure 3.**
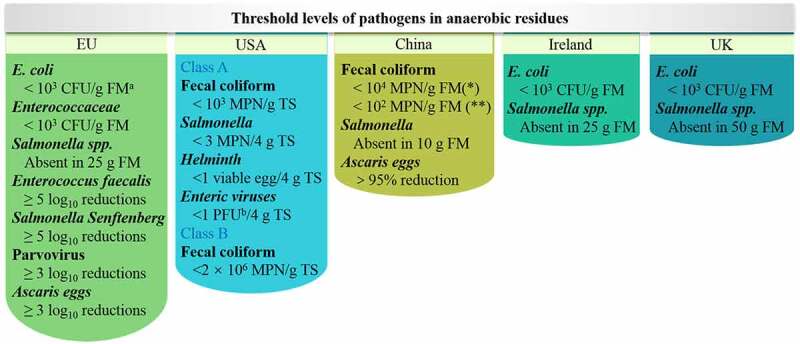
Threshold levels of pathogens in anaerobic residues.

Many countries have set biosafety regulations for pathogenic bacteria in AD [25]. And the standards vary in regulations from country to country. The indicator pathogenic organisms and their biosafety standards are shown in Figure 3. In the EU, Ireland, and the UK, *E. coli* is required to be less than 10^3^ CFU/g fresh matter, while in the USA and China, it is that fecal coliform rather than *E. coli* used as an indicator pathogen, and the requirement is less than 10^3^ and 2 × 10^6^ MPN/g TS in the USA’s Class A and Class B standards, respectively. While in China, fecal coliforms are required to be less than 10^4^ MPN/g fresh matter in ambient and mesophilic AD (MAD) systems and less than 10^2^ MPN/g fresh matter in thermophilic AD (TAD) systems. *Salmonella* is required to be absent in fresh matter or total solid among the enumerative countries. One peculiarity of the EU standards is that they require log_10_ reductions rather than specific amounts for *Salmonella Senftenberg* (≥ 5 log_10_ reductions), Parvovirus (≥ 3 log_10_ reductions), and *Ascaris eggs* (≥ 3 log_10_ reductions). The difference in national standards makes it difficult to compare horizontally.

a, FM, fresh matter; b, PFU, plaque-forming unit; *, in ambient AD and MAD; **, in TAD.

Only when the standard requirements are met can the digestate be allowed for land application. However, a potential problem is that even if the level of indicator pathogens meets the requirements, it cannot be directly interpreted as the absence of potential disease risk, because there may be other non-indicator pathogens. Therefore, it is necessary to strengthen and extend the study of pathogen indicator bacteria and improve the sanitary evaluation method.

### 3.2. Spore-forming pathogens

Bacterial species have different coping mechanisms with selective harsh environmental conditions. One of the most common coping mechanisms for bacteria is producing resistant forms called ‘spores’ to withstand extreme conditions of starvation, acidity, temperature, and desiccation. And those bacteria are called spore-forming bacteria. Typically, gram-positive bacteria can produce intracellular spores called endospores as a survival mechanism. Endospores are highly retractile and thick-walled structures formed inside the bacterial cells [26], which are the dormant form of vegetative bacteria, carrying all the genetic material as is found in the vegetative form, but no an active metabolism. They are highly resistant to physical and chemical influences and act as a means of survival during hard times. When the environmental conditions turn favorable, endospores germinate back into vegetative cells (an active bacterial cell that undergoes metabolism). Bacteria can survive for many years in this dormant stage in the normal environment because of their spore characteristics.

*Bacillu* (aerobic) species as well as *Clostridium* (anaerobic) species are the most common spore-forming bacteria. *Bacillus* is a common source of contamination in food commodities. The ability of such bacteria to adapt to differences in temperature, pH, and nutrient sources promotes their multiplication in foods and their ability to cause food spoilage [27]. *B. cereus* is a member of the *Bacillus* species and it is facultative aerobic bacteria, growing within the temperature range of 10–48°C (optimum temperature is 28–35°C). *Clostridium* is gram-positive bacteria and only sporulates anaerobically. *Clostridium botulinum, Clostridium perfringens*, and *Clostridium difficile* are species of widespread concern due to their pathogenic nature. *Clostridium botulinum* produces botulinum toxin and causes botulism. Infections with *Clostridium perfringens* can cause necrosis and gangrene. And *Clostridium difficile* can cause a series of diseases such as clinical manifestations with acquired diarrhea and pseudomembranous enteritis by producing multiple toxins [28]. Table 3 summarizes the characteristics of the three *Clostridium* species.

**Table 3.** The characteristics of *Clostridium botulinum, Clostridium perfringens*, and *Clostridium difficile.*

Types	Characteristics
*Clostridium botulinum*	Toxin types: A, B, C, D, E, F, G. Human botulism (A, B, E, F); Botulism in animals (C, D). Food poisoning: botulism. Incubation period: 8 hours to 8 days. Dose: 0.005–0.1 µg (proteolytic); 0.1–0.5 µg (non-proteolytic) for lethal. Symptoms: generalized muscular weakness; headache; dizziness; visual disturbances; nausea; vomiting; respiratory failure. Unique characteristic: strict anaerobic bacteria; heat labile toxin; produce neurotoxin. Inhibitory pH: 4.6 (A, B, F); 5.0 (B, E, F). Temperature range: 10–48°C (A, B, F); 3.3–45°C (B, E, F). D of spores: D_100_: 25 min (A, B, F); <0.1 min (B, E, F).
*Clostridium perfringens*	Toxin types: A. Food poisoning: Foodborne toxic infection. Incubation period: 8–22 hours. Dose: >10^5^ cells/g for infective. Symptoms: diarrhea, severe abdominal pain, nausea (occasionally). Unique characteristic: strict anaerobic bacterium; produce toxins (Enterotoxin; Toxin production in the digestive tract is associated with sporulation). Inhibitory pH: 5.0. Temperature range: 15–50°C (optimum 43–45°C). D of spores: D_95_: 1.3–6.4 min.
*Clostridium difficile*	High prevalence of *C. difficile* in animals. *C. difficile* in foods: further research is needed. *C. difficile* is a major cause of illness. Initially recognized as a hospital pathogen; Now recognized as an important cause of severe community-acquired infections. The source of community-acquired *C. difficile* is yet to be established. Foodborne is one route considered.

D of spores, decimal reduction time of spores; D_100_, D at 100°C; D_95_, D at 95°C.

The cells of *Bacillus* and *Clostridium* are sensitive to thermophilic temperature but the spores are highly heat-resistant. It was reported that pathogens such as *Salmonella* and *Mycobacteria paratuberculosis* were inactivated within 24 h in TAD while weeks and even months were needed in MAD [29]. However, spores of *Clostridium perfringens* type C and *Bacillus cereus* in cattle and pig slurry were not inactivated in MAD (35°C) or TAD (53°C) [30]. Pasteurization heating is insufficient to destroy the *Clostridium perfringens* spores in cow manure. In the batch AD experiment of cow manure, *Clostridium perfringens* was reduced by 1.35 log_10_ reductions at 35°C after 15 days, and <1 log_10_ reduction was observed at 55°C (2 days) and 70°C (1 day) [31]. It was reported that the decimal reduction time (DRT) of vegetative cells was 33.2 min at 50°C and 1 min at 60°C for *Bacillus cereus*,16.3 min at 55°C and 0.9 min at 65°C for *Clostridium perfringens*, respectively; while the DRT of *Bacillus cereus* spores was 32.1 min at 85°C and 2.0 min at 95°C, and the DRT of *Clostridium perfringens* spores was 34.2 min at 90°C and 2.2 min at 100°C [32]. In most studies about AD, 0–1.8 log_10_ reductions were observed at the temperature of 24–55°C and HRT of 7–56 days [25]. Sometimes the concentrations of *Clostridium perfringens* in the anaerobic residues were even higher than those in the substrates, with 0.12–1.3 log_10_ increases at the HRT of 15–90 days [33–36]. The final concentrations in all of them were above 3 × 10^3^ spores/g dry matter (DM).

### 3.2. Overlook of VBNC pathogens

**Figure 4.**
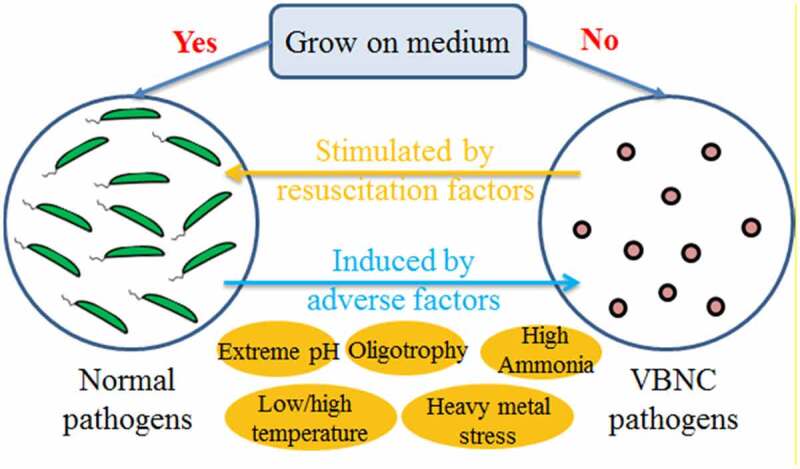
The relationship between normal pathogens and VBNC pathogens.

VBNC state is a dormant-like state of bacteria, and many species of bacteria can enter this state when suffering stress conditions. It was considered a survival strategy for bacteria to avoid adverse conditions. This phenomenon was discovered by Xu et al. when they studied *E. coli* and *Vibrio cholera* in 1982 [37]. Many factors including low or high temperature, extreme pH, oligotrophy, disinfectant, and high-energy rays can induce bacteria into VBNC state [38,39]. Compared with the normal growing cells, the metabolic activity of the VBNC bacteria was greatly reduced, the ability to divide and prolifize was decreased, and the ability to form colonies on the medium was lost. Most of the bacteria such as gram-negative bacteria which do not produce spores can enter the VBNC state to resist the external environmental pressure, to achieve the purpose of continued survival. Up to now, more than 80 species of bacteria have been reported to be able to enter the VBNC state under adverse environmental conditions. Pathogens in animal manure, such as *E. coli, Salmonella, Shigella, Enterococcus, Listeria*, are potential VBNC bacterial during AD. VBNC pathogens usually do not cause diseases, but they still keep virulence. They can resuscitate to culturable state and recover their pathogenicity when conditions become favorable. It was reported that *E. coli* entered the VBNC state in the MAD and TAD of sludge. During the subsequent centrifugation dehydration, culturable *E. coli* were found to revive and multiply [40]. The relationship between normal pathogens and VBNC pathogens is shown in Figure 4.

The count of E. coli in cow dung was measured by MPN and quantitative polymerase chain reaction (qPCR), and the result of the latter was 100 times higher than that of the former, indicating that most *E. coli* was nonculturable [15]. There are more reports about the VBNC of pathogenic bacteria in sludge AD. In the TAD of sludge, the count result of *E. coli* obtained by the traditional culture method was 20 CFU/g dry solids (DS), while that obtained by competitive PCR (cPCR) was 7 × 10^5^ CFU/g DS. The difference showed the existence of the VBNC [41]. It was found that the total viable count of *E. coli* in sludge effluent was similar to the feed concentration of 9–10 log_10_ MPN/g DS through the MAD (35°C), TAD (55°C), and temperature-phased anaerobic digestion (TPAD) (55°C + 35°C). Further study found that the culturable *E. coli* increased by 2, 2, and 4 orders of magnitude immediately after dewatering for the MAD, TAD, and TPAD processes, respectively. The results of molecular quantifying methods indicated that the sudden increase of culturable pathogen content after dewatering is actually the reactivation of *E. coli* from the VBNC state to the culturable state [40]. Due to the existence of pathogen VBNC state, AD thus mainly alters the culturable state of pathogens rather than killing them, which greatly limits the efficiency of AD in inactivating pathogens. The current evaluation of the inactivation effect of pathogens by AD is based on cultivable pathogens. However, VBNC pathogens cannot form colonies on the culture medium, resulting in false-negative test results and ultimately overestimating the inactivation performance. The true inactivation rate of viable pathogens in AD should be considered.

### 3.3. Persistent pathogens phenomenon

Persister cells are often a component of a stringent response of the microorganism to unfavorable conditions, especially antibiotics, which are among the most commonly used drugs worldwide due to their capacity of killing or inhibiting the growth of bacteria to fight bacterial infections [42]. They are dormant variants of regular cells that form stochastically in microbial populations and are highly tolerant to antibiotics. Persister cells gain their resistance property protecting them from toxic compounds acting by entering a state of dormancy. In this state, they are metabolically less active cells. The remaining metabolic activities are mostly focused on energy production [43]. However, they do not grow or die in the presence of antibiotics. In the absence of antibiotics stress, persister cells can return to sensitive and active growth phenotypes. Persister cells may be associated with chronic or recurrent infections [44]. Bacteria with persistence have been reported, including *Mycobacterium tuberculosis, Salmonella typhi, Chlamydia, Brucella, E. coli*, and *streptococcus* [45,46].

In AD, the prevalence of antimicrobial resistance among bacteria isolated from influents and effluents has been reported. Antibiotic-resistant pathogens may proliferate through the environment and allow the spread of resistance genes through bacterial genetic recombination, thus influencing human and animal antimicrobial chemotherapy [47]. A persistence of drug-resistant bacteria in thermophilic co-digestion of dairy manure and waste milk at 55°C until the end of the process was observed by Beneragama [48].

## 4. Challenges in establishing strong inactivation AD process

The inactivation efficiency of pathogens is affected by many factors, including pathogen type, intermediate products, and operating conditions, especially temperature [25]. The combined effects of these factors eventually led to the inactivation of pathogens. However, the resistance of pathogens causes obstacles to the effective inactivation of pathogens. In addition, it is reported that it’s more difficult to reduce pathogens for treatment on larger scales (e.g., full scale biogas plants) than that on laboratory scales [49]. Therefore, how to strengthen the inactivation of pathogens in practical engineering should be further discussed.

### 4.1. Mesophilic vs thermophilic process

AD generally can be divided into TAD (50–55°C) and MAD (30–42°C). Most pathogens survive longer at mesophilic temperatures than at thermophilic temperatures. As the temperature increases (from 37 to 70°C), the fluidity and permeability of the cell membrane increase, which allows toxic chemicals to diffuse more rapidly into the cytoplasm and inhibits cell growth [50]. In a mesophilic (35°C) small-scale AD, average T_90_ values, which is defined as the time for bacterial concentration to decrease by 90%, were 1.8 days for *E. coli*, 2.0 days for *Salmonella dublin*, and 0.9 days for *Staphylococcus aureu*. In a thermophilic (53°C) small-scale AD, T_90_ values of 0.4 h were determined for *E. coli*, 0.6 h for *Salmonella dublin*, and 0.5 h for *Staphylococcus aureus* [30]. TAD has advantages over MAD in inactivating pathogens, but considering energy consumption, process stability, and diversity of anaerobic microorganisms, MAD is more widely applied to practice [51]. Given this, the AD process should have greater innovation in inactivating pathogens.

### 4.2. Is multi-stage worthy for application?

Worldwide, single AD is more widely used than multi-stage process despite the latter having many advantages. Due to the characteristics of the substrate, pretreatment is needed to improve biogas production in single AD, and temperature is the parameter that had the greatest effect during AD [52]. Pasteurization as a typical heat-treatment method, can be regarded as a stage in a multi-stage AD. In Swedish biogas plants, the undigested substrate is heated to 70°C in 60 min in a separate batch-wise step before AD. Germany and Austria also recommend that animal and poultry manure should be sterilized at 70°C for 20 or 30 min before entering AD tanks, to ensure the safety of biogas engineering [53]. In Denmark, pasteurization (70°C in 60 min) may be replaced with a ‘similar method’ (a longer time at a lower temperature). This is combined with thermophilic or mesophilic digestion with regulated temperature and time [13], called TPAD. However, pasteurization is always different from the first stage of multi-stage AD, and it is still not commonly used in underdeveloped areas or countries.

In the first stage of multi-stage AD, a high temperature or/and high VFAs concentration in the environment, which are considered as the main factors of pathogens inactivation, can be created by adjusting process parameters. VFAs are essential intermediates produced in the acidogenesis and acetogenesis step when organic materials are degraded during the AD process [54], and free VFAs and ionized VFAs are the two forms of VFAs. Free VFAs are considered more toxic to pathogens than ionized VFAs, as they are lipophilic and can freely permeate the cell membrane [55]. The toxicity of VFAs is dependent on the pH, which affects the distributions of free and ionized VFAs, thus affecting pathogen inactivation indirectly. Under the condition of low pH, VFAs mainly exist in a molecular state, which indicates that the high VFAs concentration and low pH are more conducive to inactivating pathogens. In an anaerobic digester for cattle dung with VFA levels of 5000 mg/L and pH 6.0, *Salmonella typhi* was completely eliminated within 12 d, whereas 26 d were required in the digester with VFA of 100 mg/L and pH 6.8. And T_90_ values for the two digesters were 2.44 d and 4.80 d, respectively [56]. VFAs mainly include acetic, propionic, isobutyric, butyric, isovaleric, valeric, and caproic acid [57]. Generally, the inhibitory effect of VFAs on pathogens increases with the increase of the organic acid chain length. A greater inactivation effect of n-caproic acid against *Ascaris eggs* compared with n-butyric acid was reported [58]. Jiang et al. reported the inhibitory effect of VFAs against *Salmonella* was in the order of valeric acid > isovaleric acid ≥ propionic acid > butyric acid ≥ isobutyric acid > acetic acid [59]. The first stage of multi-stage AD can effectively inactivate pathogens due to high temperature or VFA, and the second stage can realize the stable production of methane. Therefore, multi-stage AD should be considered to be popularized.

### 4.3. Tradeoff between energy production and pathogens inactivation

Bacterial inactivation due to temperature is related to time. In general, the longer the hydraulic retention time (HRT), the better the inactivation effect of pathogens. At the same temperature (35°C), a biogas plant with a longer HRT of 40 d was detected a significantly higher reduction of *Campylobacter* (87.4%) than 74.3% that in a biogas plant with HRT of 30 d [60]. However, the extension of HRT reduces the volumetric gas production rate and energy production efficiency, which becomes contradictory with the sterilization effect. In addition, some studies have also pointed out that prolonged retention time has no significant effect on some pathogens. When the HRT was increased from 21 d to 41 d at MAD (39°C) of pig manure, the inactivation amount of *E. coli* increased slightly but not significantly, and the concentration was between 2.0 and 2.5 log CFU/g [61]. When the HRT was increased from 7.4 d to 46.9 d, the reduction of *E. coli* in AD is in the range of 0.19 log_10_ to 2.99 log_10_, which is no correlation with HRT [62]. Therefore, retention time is not a direct factor affecting the inactivation effect of pathogens and it works alongside other factors to inactivate pathogens. The specific inactivation mechanism needs to be further studied.

### 4.4. Double-edged sword of ammonia for fermentation and hygienization

Ammonia is the intermediate product of AD, which is released due to the degradation of organic molecules containing N such as proteins. Literature has reported the positive effect of ammonia on pathogen reduction in the mesophilic process at a total ammonia (TAN) concentration of 6–9 g/L [63]. In addition, free ammonia (FA, NH_3_) is considered more toxic to pathogens than ammonium ion (NH_4_^+^) due to its lipophilic property, which can pass the cell membrane freely. Temperature, pH, and TAN determine the presence of FA, substantially [64].

In a mesophilic anaerobic digester for pig manure, pathogens (Fecal coliform, *E. coli*, fecal streptococci, and *Enterobacteriaceae*) content and toxic-NH_3_ content were significant negative correlations [33]. The NH_3_ concentration of 644 mg N/L (46 mM) caused 5 log_10_ reductions on *Salmonella Typhimurium* and *Enterococcus faecalis* after 1.6 and 4.2 days, respectively [63]. There was a complete *Salmonella* reduction when the toxic-NH_3_ content was more than 560 mg NH_3_–N/L (40 mM) after 6 h, but 2520 (180 mM) mg NH_3_–N/L was needed to obtain >5 log_10_ reductions for *E. coli O157:H7* after 6 h [65]. It indicated that the higher concentration of ammonia nitrogen, the better the inactivation effect of pathogens.

However, the high concentration of ammonia nitrogen in an AD system will inhibit methanogenesis to some extent [66]. It is generally believed that the lower limit concentration of ammonia nitrogen inhibiting methane fermentation is 3.0–4.0 g/L. But a successful biogas plant running under a much higher ammonia nitrogen level was reported [67]. FA is freely membrane-permeable, which has been suggested to be the main reason for the inhibition [68]. It was reported that FA concentrations of 150–400 mg/L are a cause of inhibition in TAD during the appraisal from a previous study [69]. A 50% inhibition of methanogenesis was observed during TAD of pig manure at a FA concentration of 1450 mg/L [70]. Therefore, in the anaerobic treatment of animal manure with high nitrogen content (such as chicken manure), high ammonia nitrogen has a positive effect on the inactivation of pathogens, but the possible adverse effects of high ammonia nitrogen on methane fermentation should also be considered. When reasonable organic load rate (OLR) and HRT were set, the AD methanogenesis system can also operate normally under a high ammonia nitrogen concentration of 6000 mg/L [71].

## 5. Conclusions and prospects

AD is an effective way to realize the reduction treatment and resource recovery and utilization of animal manure, but it cannot completely inactive pathogenic bacteria. Pathogens have coping mechanisms with adverse conditions to survive to the highest extent, providing spore-forming pathogens and VBNC pathogens as the main forms. Conventional AD processes are thus difficult to effectively inactivate those patognes. High temperature, high VFAs, long retention time, and high ammonia concentrations have positive effects on inactivating pathogens. Those conditions can be achieved in an AD process and may be used to deeply inactivate pathogens. Multi-stage AD was consequently proposed as a potential approach to meet the objectives of high efficient methanogenesis and hygienization in AD.
